# Pairing of integrins with ECM proteins determines migrasome formation

**DOI:** 10.1038/cr.2017.108

**Published:** 2017-08-22

**Authors:** Danni Wu, Yue Xu, Tianlun Ding, Yan Zu, Chun Yang, Li Yu

**Affiliations:** 1 State Key Laboratory of Biomembrane and Membrane Biotechnology, Tsinghua University-Peking University Joint Center for Life Sciences, School of Life Science, Beijing 100084, China; 2 Institute of Biomechanics and Medical Engineering, School of Aerospace, Tsinghua University, Beijing 100084, China.

## Dear Editor,

Recently we reported the discovery of migrasome, a new organelle of migrating cells^1^. Migrasomes are large vesicles that grow on the tips or intersections of retraction fibers at the rear of migrating cells. Following cell migration, the retraction fibers eventually break and the migrasomes become detached. The migrasomes and their contents, including cytosolic components and vesicles of unknown origin, are released into the extracellular space —— a process we named as migracytosis. We speculated that migracytosis may play important roles in cell-cell communication.

Integrins are a large family of transmembrane receptors that connect cells to the extracellular matrix (ECM). Integrins function as heterodimers composed of α and β subunits; there are 18 α and 8 β integrins in mammals^2^. Different integrins bind to different ECM proteins^2^. For example, integrin α5β1 specifically binds to fibronectin, α3β1 to laminins, and α1β1 and α2β1 to collagens^3^.

**Figure 1:**
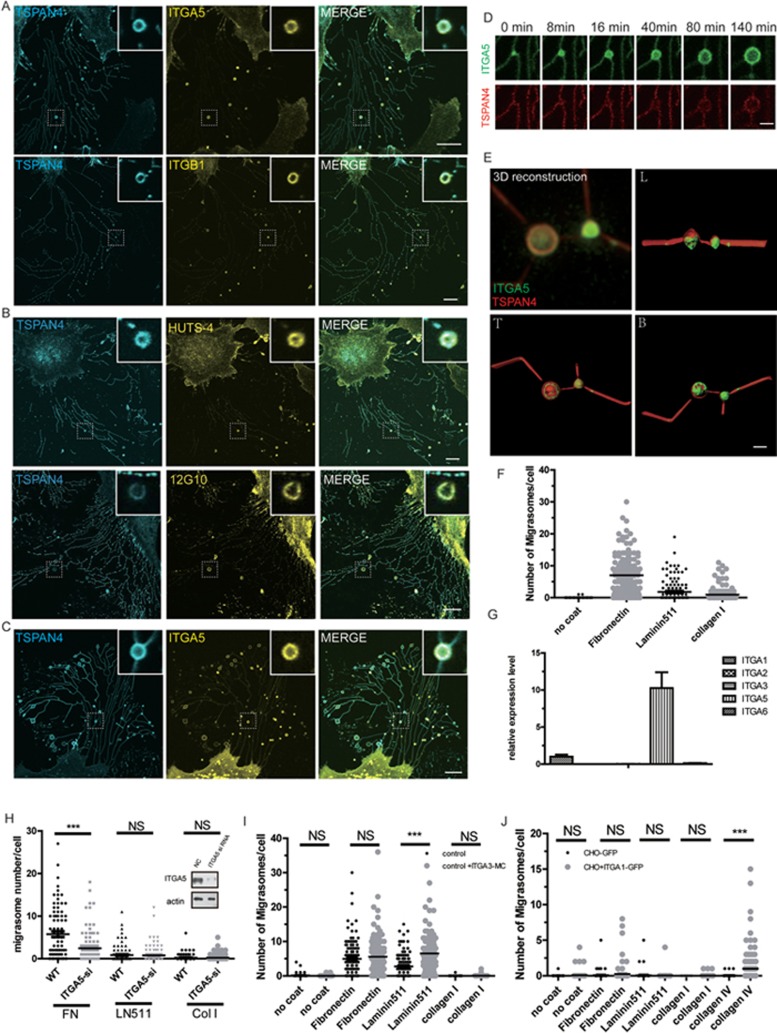
**(A)** Immunofluorescence (IF) staining of integrin α5 (ITGA5) and β1 (ITGB1) in MGC803 cell line overexpressing TSPAN4-GFP. Scale bar, 10 μm. **(B)** IF staining of active integrin β1 (HUTS-4 or 12G10) in TSPAN4-GFP-overexpressing MGC803 cells. Scale bar, 10 μm. **(C)** Live-cell images of NRK cells transfected with TSPAN4-GFP and integrin α5-mCherry. Scale bar, 10 μm. **(D)** NRK cells were co-transfected with TSPAN4-mCherry and integrin α5-GFP, and time-lapse images were taken every 8 min. Scale bar, 1 μm. **(E)** 3D reconstitution of TSPAN4-mCherry and integrin α5-GFP on migrasomes and retraction fibers. T, top view; L, lateral view; B, bottom view. Scale bar, 2 μm. **(F)** TSPAN4-GFP-expressing NRK cells were cultured on different ECM proteins (fibronectin, laminin 511 and collagen I), and the number of migrasomes formed on each was counted. **(G)** The expression level of different integrin α subunits in TSPAN4-GFP-expressing NRK cells was analyzed by qPCR. **(H)** TSPAN4-GFP-expressing NRK cells were transfected with control or ITGA5-targeting siRNA for more than 24 h, then plated on the FN-, LN511- or Col I-coated chamber overnight, and confocal pictures regarding migrasome number for each cell were taken. The western blotting analysis (the right panel) showed the knocking down efficiency. **(I)** NRK cells expressing TSPAN4-GFP alone (control) or TSPAN4-GFP with mCherry-tagged integrin α3 were cultured on different ECM proteins and the number of migrasomes per cell was counted. Data were analyzed with two-tailed *t*-tests (GraphPad Prism 5); ^***^P < 0.001. **(J)** CHO cells were transfected with pEGFP-N1 (control) or integrin α1-GFP and seeded into chambers coated with different ECM proteins, and the number of migrasomes per cell was counted. Data were analyzed with two-tailed *t*-tests (GraphPad Prism 5); ^***^P < 0.001.

When cells migrate away, migrasomes adhere to the place where they are formed, which indicates that something holds the migrasome at its formation site. Mass spectrometry analysis revealed integrin α5β1 is enriched on migrasomes (unpublished data), suggesting this integrin is a possible candidate for the molecule that holds the migrasome on the ECM. To testify this hypothesis, we used MGC803 cells expressing TSPAN4-GFP that labels migrasomes, and stained the cells with antibodies against integrin α5 and β1. We found that both integrin α5 and β1 were enriched in migrasomes (Figure 1A). Moreover, using two antibodies which specifically recognize different epitopes of activated integrin β1 (clone HUTS-4 and 12G10), we found migrasome-localized β1 was in its activated ligand-binding state (Figure 1B), which indicates that the migrasomal integrin binds the ECM.

To study the migrasomal integrin in more detail, we transfected integrin α5-GFP into normal rat kidney cells expressing TSPAN4-mCherry (NRK-TSPAN4-mCherry). We found that integrin α5-GFP was highly enriched in migrasomes, whereas there was relatively less integrin α5-GFP on retraction fibers (Figure 1C). Time-lapse analysis revealed that during migrasome growth, small integrin-positive puncta first appeared on retraction fibers, and these integrin-positive puncta grew gradually and soon became TSPAN4-positive migrasomes (Figure 1D). Thus, the formation of integrin puncta appears to specify the site of migrasome formation.

Migrasomes are spherical structures, so if integrin molecules adhere migrasomes to the ECM, they must be located at the bottom of the migrasome. To test this hypothesis, we checked the 3D distribution of integrin α5 and TSPAN4 on migrasomes. We found that integrin α5 was mainly enriched on the bottom side of migrasomes while TSPAN4 was on the upper side (Figure 1E). Similarly, TIRF microscopy imaging revealed that endogenous integrin α5 and β1 were enriched on the bottom of migrasomes (Supplementary information, Figure S1A and S1B). Taken together, these data on the spatio-temporal distribution pattern of integrin on migrasomes further support our hypothesis that integrin α5β1 adheres migrasomes to the ECM and may play important roles in migrasome formation.

Focal adhesions (FAs) are the sites that cells are linked to the ECM. Integrins are highly enriched in FAs^4^. We wondered whether the integrin-enriched regions on migrasomes are FAs. To test this idea, we co-transfected cells with various FA markers (paxillin, vinculin or zyxin)^5^ and TSPAN4. We found that none of these FA markers was localized on migrasomes (Supplementary information, Figure S2A-S2C). Moreover, the average lifetime of migrasomes is about 200 min, whereas the FAs are much more dynamic, with the lifetime of < 1 h (Supplementary information, Figure S2D). These data suggest that the integrin-enriched site on the bottom of the migrasome is not an FA. Interestingly, substrate-attached materials, which are cellular feet that remain on substrates after the treatment of adherent cells with EGTA, also contain integrins but not FA components^6^.

Different integrins bind and adhere to different ECM proteins. We reasoned that migrasome formation may depend on the matching of specific integrin-ECM pairs. To test this hypothesis, we cultured TSPAN4-GFP-expressing NRK cells (NRK-TSPAN4-GFP) on different ECM proteins. We found that NRK-TSPAN4-GFP cells produced many more migrasomes on cover glasses coated with fibronectin than with laminin 511 or collagen I, whereas very few migrasomes formed on non-coated cover glasses (Figure 1F). Next, we checked the expression level of various integrins in NRK-TSPAN4-GFP cells, including integrin α1 and α2 that bind collagen I and IV, α3 and α6 that bind laminin 511, and α5 that binds fibronectin. We found that NRK-TSPAN4-GFP cells expressed much higher levels of α5 than other integrins (Figure 1G). Furthermore, knockdown of *ITGA5* that encodes α5 impaired the formation of migrasomes on cells cultured on fibronectin, but not on other ECMs (Figure 1H). Thus, migrasome formation on NRK-TSPAN4-GFP cells is likely dependent on integrin α5-fibronectin pairing.

In NRK-TSPAN4-GFP cells, the expression level of integrin α3 is very low. We overexpressed integrin α3-mCherry in NRK-TSPAN4-GFP cells, and found that it significantly enhanced migrasome formation on cover glasses coated with laminin 511, but not on cover glasses covered with other ECM proteins (Figure 1I). Due to the reason that GFP-tagged integrin α1 does not express well in NRK-TSPAN4-GFP cells, we used CHO cells to test whether integrin α1 can promote migrasome formation on paired ECM protein. Similarly, overexpression of GFP-tagged integrin α1 in CHO cells enhanced the formation of migrasomes on collagen IV, which has the highest binding affinity to integrin α1, but not on other ECM proteins (Figure 1J). Furthermore, overexpression of integrin α1 and α3 increased cell spreading and migration on their respective ECM partner protein (Supplementary information, Figure S3). This implies that integrin α3 and α1 may affect migrasome formation by regulating cell migration and adhesion. Similarly, overexpression of α3 and α1 in MGC803 cells, which have low endogenous levels of α3 and α1, also enhanced the formation of migrasomes on the respective ECM partner protein, but not on unmatched ECM proteins (Supplementary information, Figure S4). Based on these data, we conclude that the pairing of integrin with its specific ECM partner is a determinant for migrasome formation.

Migrasome formation depends on cell migration^1^. Formation of retraction fibers requires adherence of at least a portion of the retraction fiber to the cell surface to provide a tethering point, which enables the retraction fiber to be pulled from the trailing end of the cell as the cell migrates. The fact that integrins are enriched on the bottom of migrasomes, and that migrasomes are largely stationary while retraction fibers are often swaying around (Supplementary information, movie S1) indicates that the migrasome is the point of adhesion which tethers the retraction fibers to the ECM. Thus, integrins may play dual roles in migrasome formation: the integrins on the cell body enable the cell to migrate, whereas the integrin on the migrasome provides the adhesion for retraction fiber tethering.

In this report, we found that activated integrin is highly enriched on the bottom of migrasomes, and we further demonstrated that the expression level of integrin affects migrasome formation. Finally, we showed that the correct pairing of integrin with its specific ECM partner protein is a determinant for migrasome formation.

There are 18 α and 8 β integrins in mammals and their expression is subject to various regulatory mechanisms. Different integrins bind different ECM proteins^2^, and each ECM protein has a specific spatial and temporal distribution pattern in a given organism. Thus, our finding that pairing of integrins with their specific ECM partner proteins is a determinant for migrasome formation may emerge as an important principle for determining where and when migrasomes can be generated *in* *vivo*.

So far, the detection of migrasomes has depended on TSPAN4, a marker we identified in our original paper. In this regard, integrins may serve as more specific markers for migrasomes (Figure 1A-1C and Supplementary information, movie S2). While both TSPAN4 and integrins are highly enriched on migrasomes, unlike TSPAN4 that is also abundant on retraction fibers, integrins are only present at very low levels on retraction fibers. Excellent integrin antibodies are available commercially, and thus they may emerge as important tools for studying migrasomes *in vivo*.

## Supplementary Information

Supplementary information Supplementary information Figures S1–S4 and Data S1

Supplementary information, Movie S1 NRK cells expressing TSPAN4-GFP were cultured on fibronectin coated chamber overnight and then were taken movie by every 200 second.

Supplementary information, Movie S2 NRK cells expressing TSPAN4-Cherry red and integrin α5-GFP were culture on fibronectin coated chamber and observed by time lapse microscopy. Green: integrin α5-GFP. Red: TSPAN4- mCherry; Time interval, 8 minutes.
