# Supplementary material for: Pairing of integrins with ECM proteins determines migrasome formation
Source: Cell Res. 2017 Aug 22;27(11):1397–400. doi: 10.1038/cr.2017.108 (PMC5674152; doi:10.1038/cr.2017.108)
Supplement: Supplementary information — Figures S1–S4 and Data S1 [file cr2017108x1.pdf]

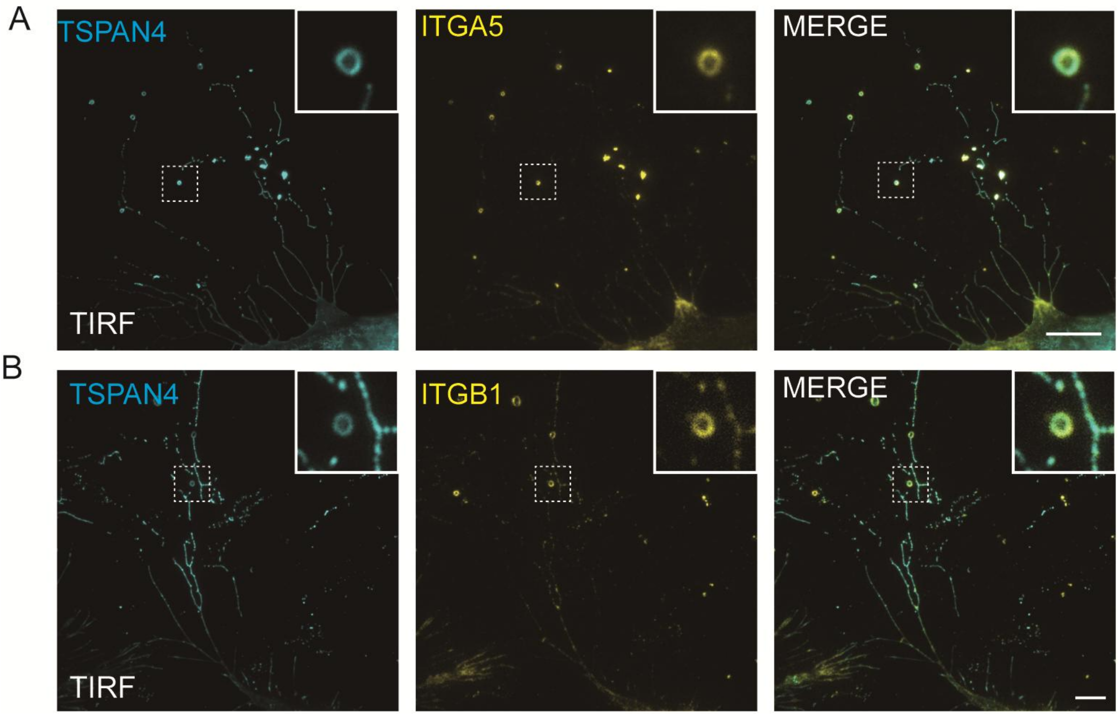
A
TSPAN4
ITGA5
MERGE
TIRF
B
TSPAN4
ITGB1
MERGE
TIRF

Supplementary information, Figure S1. Endogenous integrin α5β1 is enriched on the bottom of migrasomes. (A) TIRF image showing the staining of endogenous integrin α5 (ITGA5) in MGC803 cell line overexpressing TSPAN4-GFP. Scale bar, 10 μm. (B) TIRF image showing the staining of endogenous integrin β1 (ITGB1) in the TSPAN4-GFP-overexpressing MGC803 cell line. Scale bar, 10 μm.

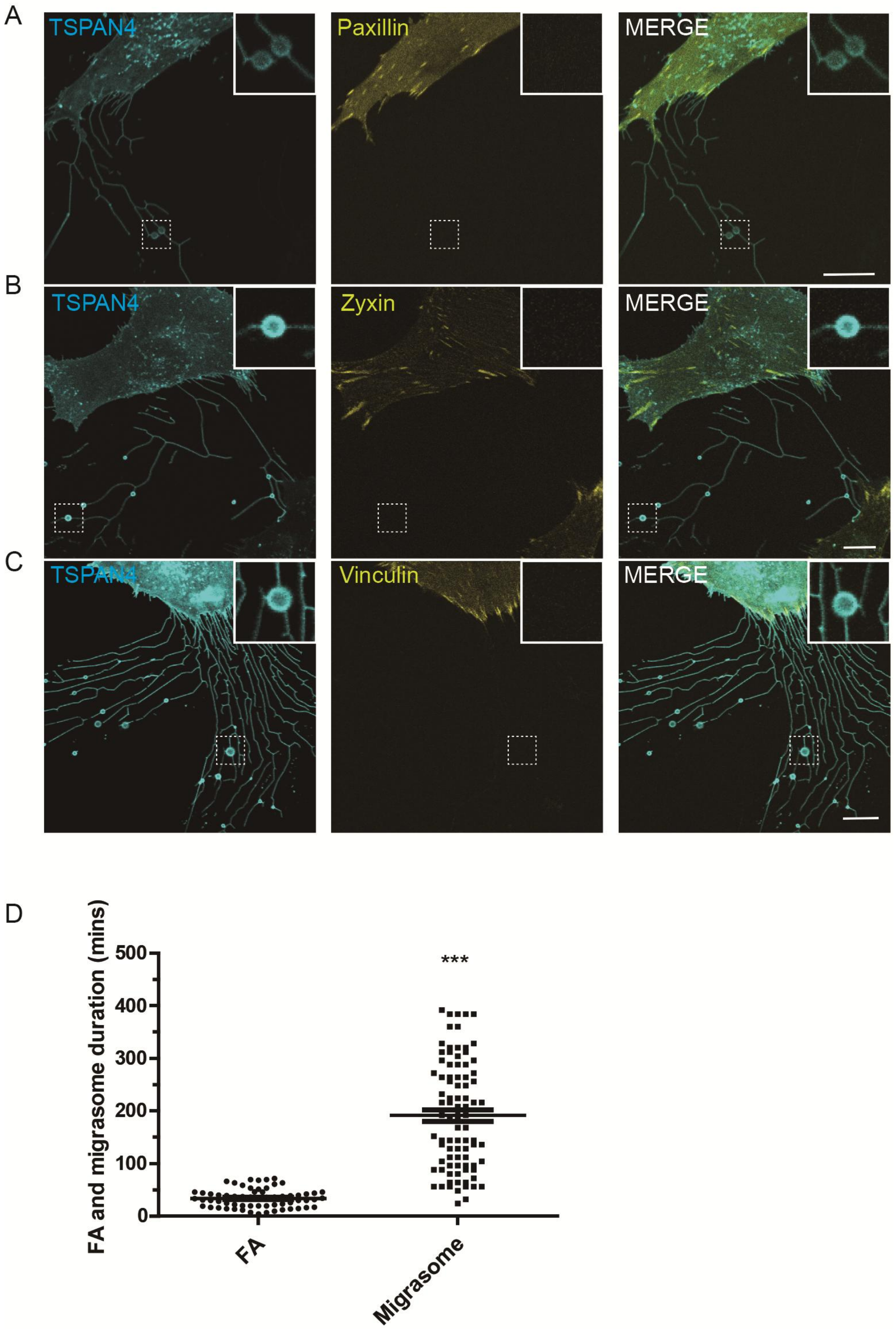
A
TSPAN4
Paxillin
MERGE
B
TSPAN4
Zyxin
MERGE
C
TSPAN4
Vinculin
MERGE
D
FA and migrasome duration (mins)
500
400
300
200
100
0
***
FA
Migrasome

Supplementary information, Figure S2. Focal adhesion proteins do not localize to migrasomes. NRK cells were co-transfected with TSPAN4-mCherry and paxillin-GFP (A) or zyxin-GFP (B) or vinculin-GFP (C). Scale bar, 10 μm. (D) Statistical analysis of FA lifetime and migrasome lifetime. Cells stably overexpressing vinculin-GFP were seeded into fibronectin-coated chambers, and time-lapse movies were taken using a TIRF microscope. Each dot represents one FA or migrasome. Horizontal bars represent the mean ± s.e.m. For statistical comparison, Student's *t*-test (GraphPad Prism 5) was employed; ***$P < 0.001$.

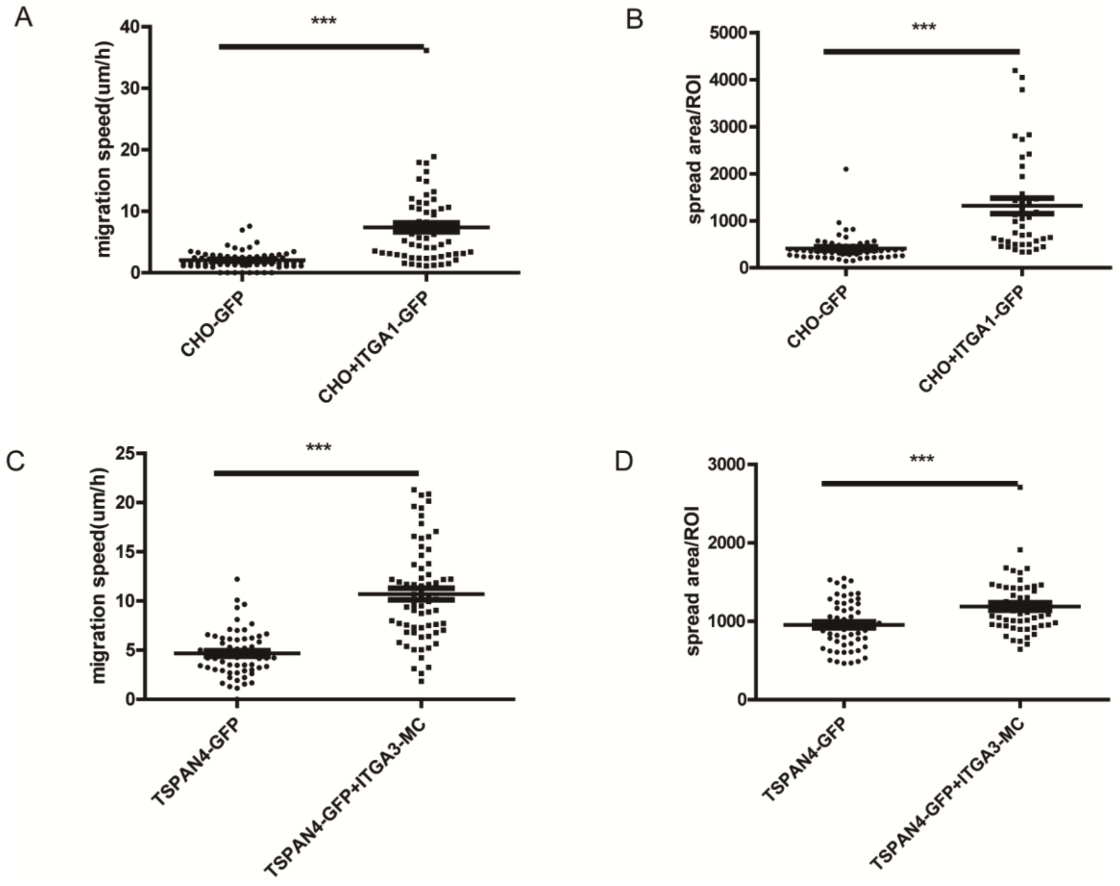
A
***
40
30
20
10
0
migration speed(um/h)
CHO-GFP
CHO+ITGA1-GFP
B
***
5000
4000
3000
2000
1000
0
spread area/ROI
CHO-GFP
CHO+ITGA1-GFP
C
***
25
20
15
10
5
0
migration speed(um/h)
TSPAN4-GFP
TSPAN4-GFP+ITGA3-MC
D
***
3000
2000
1000
0
spread area/ROI
TSPAN4-GFP
TSPAN4-GFP+ITGA3-MC

Supplementary information, Figure S3. Overexpressing integrin induces cell spreading and migration. (A) CHO cells stably expressing ITGA1-GFP or GFP were cultured on Col IV-coated chambers overnight, then a movie was taken every 5 min. The cell migration speed and cell spreading area was recorded using NIS-elements software, and the data were analyzed by Graph-Pad Prism5; ***P < 0.001. (B) Cells stably expressing TSPAN4-GFP and ITGA3-mCherry, or TSPAN4-GFP alone, were cultured on LN-511-coated chambers overnight, then a movie was taken every 5 min. The cell migration speed and cell spreading area were recorded with NIS-elements software, and the data were analyzed by Graph-Pad Prism 5; ***P < 0.001.

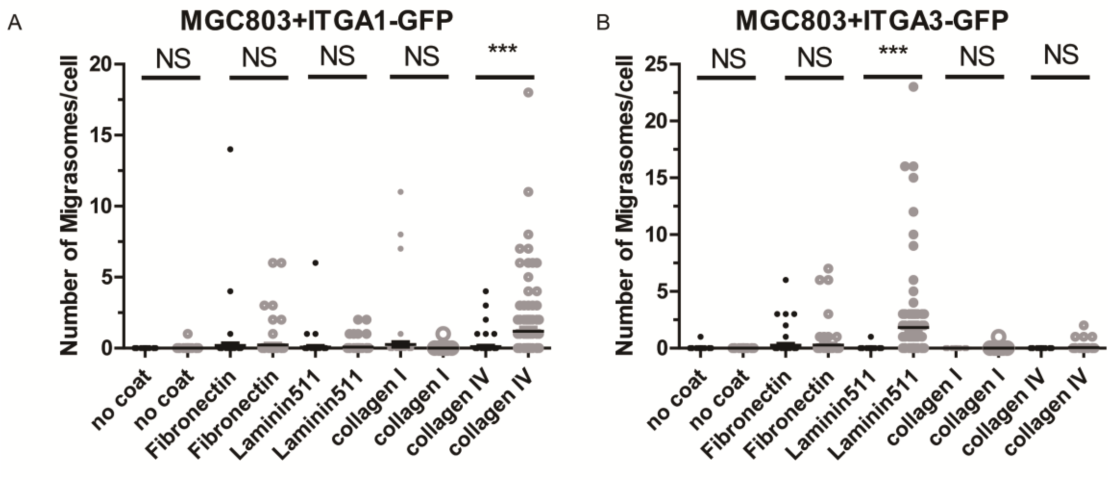
A
MGC803+ITGA1-GFP
Number of Migrasomes/cell
NS
NS
NS
NS
***
20
15
10
5
0
no coat
no coat
Fibronectin
Fibronectin
Laminin511
Laminin511
collagen I
collagen I
collagen IV
collagen IV
B
MGC803+ITGA3-GFP
Number of Migrasomes/cell
NS
NS
***
NS
NS
25
20
15
10
5
0
no coat
no coat
Fibronectin
Fibronectin
Laminin511
Laminin511
collagen I
collagen I
collagen IV
collagen IV

Supplementary information, Figure S4. Overexpressing integrin also induces migrasome formation in MGC803 cell. (A) MGC803 cells were transfected with ITGA1-GFP (grey) or control GFP (black) and plated on chambers coated with different ECM proteins. Images were then taken with a confocal microscope and the number of migrasomes in transfected cells was counted and compared with control cells. ***P < 0.001. (B) MGC803 cells were transfected with ITGA3-GFP (grey) or control GFP (black) and plated on chambers coated with different ECM proteins. Confocal images were then taken and the number of migrasomes was counted. ***P < 0.001.

Supplementary information, Data S1.

Materials and Methods

Cell culture and transfection

NRK cells were cultured in DMEM (Life Technologies) supplemented with 10% FBS (5% CO2). Cells were transfected with total of 4 μg DNA via Amaxa nucleofection using solution T and program X-001. Cells were then cultured in growth medium for further analysis.

Cells were seeded in a 6-well plate at a density of 6×10^5 per well and transfected with 100 μl serum-free Opti-MEM that contained 2 μl LipofectamineTM 2000 (Thermo Fisher Scientific), 2.5 μg plasmid. Then replace with new medium after 4 hours' transfection.

Antibodies and ECMs

Primary antibodies were purchased from the following sources: HUTS-4 (active β1-integrin, Millipore), 12G10 (active β1-integrin, Abcam), 4B7R (total β1-integrin, R&D), VC5 (α5-integrin, BD Pharmingen); for western blot, α5-integrin, #4705, Cell Signaling Technology.

Fibronectin is purchased from life Technology, laminin 511 is from BioLamina, and type I collagen is form Solarbio, and collagen IV is from ZSBG-Bio.

Plasmid construction

α5-integrin-GFP was obtained from Addgene (#15238), α3-integrin-GFP, α1-integrin-GFP paxillin-GFP, vinculin-GFP, zyxin-GFP was clone into pEGFP-N1 by Clone Express® II One Step Cloning Kit (Vazyme).

RNA transfection and Quantitative polymerase chain reaction (qPCR) analysis

Cells were transfected with 200 pmol RNAi via Amaxa nucleofection using solution T and program X-001. Cells were then cultured in growth medium for further analysis. For two rounds of transfection, cells were transfected with 200 pmol RNAi, and 48 h after transfection, cells were transfected again with 100 pmol RNAi.

Total RNA was extracted from cells using Trizol reagent (Invitrogen), and 1 μg of RNA was used as a template for reverse transcription using random primers. qPCR was performed to characterize the mRNA levels of specific genes using 2×RealStar Green Power Mixture (GeneStar) in a Roche Light 480 Real-Time PCR machine.

Immunofluorescence

Cells were washed with phosphate buffered saline (PBS), fixed in 4% paraformaldehyde for 10 min or ice-cold Methanol for 5 min, and permeabilized in 0.1% saponin for 5 min. Fixed cells were blocked with 10% FBS in PBS for 30 min, stained with 10 $\mu g ml^{-1}$ of antibody in blocking buffer for 1 h, and washed with PBS three times. Cells were then stained with secondary antibody in PBS for 1 h and washed with PBS three times.

Live-cell imaging

The night before imaging, cells were cultured in 35 mm glass-bottom dishes coated with different ECMs (10 μg/ml). Images were acquired using Nikon A1 and Olympus FV-1000 confocal microscopes. And the 3D reconstitution is made by Imaris software.

Cell adhesion and migration assays

Cells were cultured on ECM coated chamber for overnight, and then taken movie using Nikon A1 microscope, time interval is 5min. Cell spreading surface and cell track is analyzed by Nikon A1 analysis software.

Statistical analysis

Statistical analysis was performed in GraphPad Prism5. All data were obtained from independent experiments. Error bars in the figures represent the standard deviation (SD). *n* values are specified in the figure legends.
